# 11 Evaluation of doctors’ knowledge (excluding rheumatologists and pediatricians) of juvenile idiopathic arthritis

**DOI:** 10.1093/rheumatology/keac496.007

**Published:** 2022-10-07

**Authors:** Houssem Tbini, Samia Jemmali, Soumaya Boussaid, Ahlem Ben Ammou, Safa Rahmouni, Sonia Rekik, Khaoula Zouaoui, Hela Sahli, Mohammed Elleuch

**Affiliations:** 1 Department of Rheumatology, La Rabta Hospital, Tunis, Tunisia; 2 University of Tunis El Manar

## Abstract

**Background:**

Juvenile idiopathic arthritis (JIA) is the most common pediatric inflammatory arthritis. However, this disease is not fully understood by all practitioners.

**Objectives:**

The objective of this survey is to assess the level of knowledge and attitudes of doctors excluding rheumatologists and pediatricians on the management of JIA.

**Methods:**

In this cross-sectional descriptive study, an anonymous questionnaire composed of 20 questions, designed with the Google-Forms software, was sent via social networks to doctors from different specialties (excluding rheumatologists and pediatricians) and to interns and residents of the University Hospitals of Tunis.

**Results:**

A total of 100 physicians responded to the questionnaire (61 women and 39 men). The mean age was 31.01 ± 5.64 [24–51] years. Fifty-seven percent of the participants were in training, 27% worked in public hospitals and 16% in private clinics. Most participants were general practitioners (32%), followed by ophthalmologists (9%), gynecologists (7%), and internists (5%). the mean length of service was 4.99 ± 4.5 [0–22] years. Thirty-six participants affirmed they have been confronted with patients with JIA. Twenty percent of participants believed they have knowledge about JIA. Among them, 25% believed they have sufficient knowledge, the others had superficial knowledge. Thirty-four percent of participants knew the definition of the disease according to the age, 17% knew its frequency and 18% had an idea about its various sub forms. The most frequent sub form was known only by 32 participants.

Regarding the complications of JIA, only 25% of the participants thought of the macrophage activation syndrome in case of deterioration of the general state, fever, and pancytopenia. In addition, 44% of participants knew that JIA can be complicated by ocular involvement.

Concerning the presence of rheumatoid factors and anti-nuclear antibodies during JIA, 64% and 60% respectively answered that their presence was not necessary.

Concerning the care of JIA, 64% of participants referred these patients to rheumatologists, 31% to a pediatrician, and 5% took care of them themselves.

The interest of methotrexate and biological treatments were known by 48% of the participants. However, 37% of participants had no idea of the therapeutic management of JIA.

Concerning the prognosis, only 25% of the participants know the sub forms of bad prognosis. However, 48% had no idea about the prognosis of the disease.

**Conclusion:**

The level of knowledge of doctors on JIA is low to average. Training for physicians on this potentially serious disease is needed.

